# Electrolyte-Gated Graphene Field Effect Transistor-Based Ca^2+^ Detection Aided by Machine Learning

**DOI:** 10.3390/s23010353

**Published:** 2022-12-29

**Authors:** Rong Zhang, Tiantian Hao, Shihui Hu, Kaiyang Wang, Shuhui Ren, Ziwei Tian, Yunfang Jia

**Affiliations:** 1 College of Electronic Information and Optical Engineering, Nankai University, Tianjin 300350, China; 2 School of Integrated Circuit Science and Engineering, Tianjin University of Technology, Tianjin 300384, China

**Keywords:** electrolyte-gated graphene field effect transistor, Ca^2+^ detection, machine learning, regression model, calibration-free, flexible

## Abstract

Flexible electrolyte-gated graphene field effect transistors (Eg-GFETs) are widely developed as sensors because of fast response, versatility and low-cost. However, their sensitivities and responding ranges are often altered by different gate voltages. These bias-voltage-induced uncertainties are an obstacle in the development of Eg-GFETs. To shield from this risk, a machine-learning-algorithm-based LgGFETs’ data analyzing method is studied in this work by using Ca^2+^ detection as a proof-of-concept. For the as-prepared Eg-GFET-Ca^2+^ sensors, their transfer and output features are first measured. Then, eight regression models are trained with the use of different machine learning algorithms, including linear regression, support vector machine, decision tree and random forest, etc. Then, the optimized model is obtained with the random-forest-method-treated transfer curves. Finally, the proposed method is applied to determine Ca^2+^ concentration in a calibration-free way, and it is found that the relation between the estimated and real Ca^2+^ concentrations is close-to y = x. Accordingly, we think the proposed method may not only provide an accurate result but also simplify the traditional calibration step in using Eg-GFET sensors.

## 1. Introduction

Calcium is an essential inorganic element in the human body, and it plays an important role in physiological activities, such as skeletal development [1], regulation of normal cell functions [2], gene transcription [3] and so on. Inadequate or excessive intake of calcium is associated with increased risks of osteoporosis [4], urinary stone disease [5], cardiovascular disease [6], colorectal cancer [7] and hypertension [8]. Therefore, it is important to determine the concentration of Ca^2+^ in water which is an important source of calcium intake. Many techniques have been employed to detect calcium ions, including atomic absorption spectrometry [9], fluorescence detection [10] and inductively coupled plasma optical emission spectrometry [11]. However, these methods require expensive instruments and extensive sample preparation [10].

An electronic method based on electrochemistry is preferable to the currently used instant-assay conventional methods due to its operational simplicity, cost savings and suitability for real-time detection [12]. As a kind of electrochemical sensor, an electrolyte-gated graphene field effect transistor (Eg-GFET) uses graphene as the channel material. A unique ambipolar electric field effect along with high carrier mobility enable Eg-GFET to respond to target molecules very quickly and sensitively [13,14]. Traditional bioassay always relies on calibration which is the relation between output electrical signal and target ions’ concentration obtained by using single variable approaches under some fixed working condition, i.e., the voltages of gate (*V_g_*) and drain source (*V_ds_*) for Eg-GFET. These variables could be the Dirac point voltage (*V_CNP_*) shift on transfer curves with the target concentration changing at a constant *V_ds_* [15,16] or the conductance change ratio of the graphene channel at a constant *V_g_* [17] and the source-drain current (*I_ds_*) change ratio at constant *V_g_* and *V_ds_* [18].

However, one-dimensional data analyses can only reflect the sensor’s responses in a limited range which is affected by the working condition. This method makes the application of the Eg-GFET sensor strongly dependent on the calibration curves, which is a time-consuming step and becomes an obstacle of Eg-GFETs’ popularization. The emerging machine learning approaches provide ideal solutions to this problem [19]. For example, Long Bian et al. used regression tree and random forest regression (RFR) to expand the sensitive range of the Hg^2+^ carbon-nanotube-based FET sensor [20]; Hui Wang et al. introduced a multi-variable strategy to a single-walled carbon nanotubes FET sensor system to improve the selectivity for Ca^2+^ by using support vector regression (SVR) and artificial neural network [21]. There is no doubt that machine learning can be beneficial to a FET-type sensor by generating adaptable regression models to replace the conventional calibration method. We think a calibration-free data analysis can be available for Eg-GFET-based biochemical sensors with the expectation of improving accuracy.

**Figure 1:**
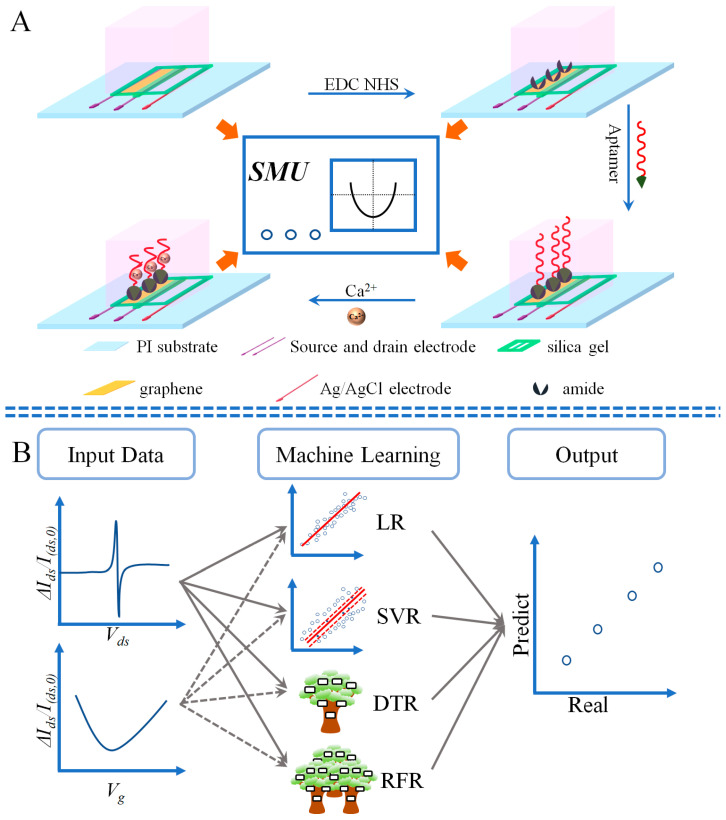
Schematic illustration of the experiments and algorithms used for calibration-free Ca^2+^ detection: (**A**) measurements during the procedure of Eg-GFETs’ functionalization by Ca^2+^ sensitive aptamer; (**B**) the machine-learning-assisted strategies for Ca^2+^ determination directly from the measured electronic signals with the utility of four algorithms, i.e., LR, SVR, DTR and RFR, respectively.

In this work, we demonstrate a machine-learning-based methodology to analyze the Eg-GFET Ca^2+^ sensors which were fabricated by using a coplanar-gate structure, self-prepared graphene ink (G-ink), a modern printing technique and the sensitive strategy of Ca^2+^ aptamer, as shown in Figure 1A. Firstly, The XPS demonstrated carboxylate groups on the graphene surface are activated by 1-ethyl-3-(3-dimethylaminopropyl) carbodiimide hydro-chloride (EDC) and N-hydroxy succinimide (NHS), then covalently linked with the amine groups modified nucleic acid strands of Ca^2+^ aptamer. The electrical properties of the as-prepared devices were carefully measured and confirmed by their typical output curves and transfer curves with the use of a source measure unit (SMU). Secondly, the linear regression (LR), SVR, decision tree regression (DTR) and RFR were studied, analyzing the measured feature curves, as shown in Figure 1B, to build the regression models and directly predict analyte Ca^2+^ concentrations from the measured electronic signals. The accuracies of the models were evaluated by using the metrics of mean square error (MSE), root mean square error (RMSE), mean absolute error (MAE) and R^2^. The results showed the best performance was acquired by using the RFR algorithm.

## 2. Materials and Methods

### 2.1. Reagents and Chemicals

The chemicals used in the experiment are listed here: graphite powder and ethyl cellulose (EC, molecular weight: 448.474) were purchased from Shanghai Minaire Chemical Technology Co., Ltd., Shanghai, China; ethanol was purchased from Shanghai Aladdin Biochemical Technology Co., Ltd., Shanghai, China; terpineol was acquired from Tianjin Jiangtian Chemical Technology Co., Ltd., Tianjin, China; phosphate buffer solution (PBS) was purchased from Beijing Solarbio Science &Technology Co., Ltd., Beijing, China. EDC and NHS were purchased from Shanghai Yuanye Biological Technology Co., Ltd., Shanghai, China; NaCl and KI were purchased from Tianjin Fengchuan Chemical Reagent Technology Co., Ltd., Tianjin, China; MgCl_2_ was purchased from Tianjin Chemical reagent Supply and Marketing Company, Tianjin, China; BaCl_2_ was purchased from Tianjin University Kewei Company, Tianjin, China. The calcium standard solution was purchased from Changzhou Tanmo Quality Inspection Technology Co., Ltd., Changzhou, China. Silver paste was purchased from Shenzhen Haori Electronic Materials Co., Ltd., Shenzhen, China. Ag/AgCl paste was purchased from Shanghai Julong Electronic Technology Co., Ltd., Shanghai, China. The calcium-detecting DNA aptamer (3′-GGGGTTTTGGGG-5′) had been modified with an amine group on the 3′ terminal and was synthesized by Sangon Biotechnology Co. (Shanghai, China).

### 2.2. Prepare Graphene Ink

G-ink was synthesized in the same way as our previous work [22]. Briefly, 2.5 g graphite powder, 0.5 g EC and 50 mL ethanol were added into a centrifuge tube and mixed thoroughly. The mixture was treated with ultrasound for 3 h and then centrifuged at a speed of 4000 rpm for 30 min. The supernatant was mixed with terpineol (*v/v* = 1:5) and treated in a 75 °C water bath for about 15 h to obtain G-ink.

### 2.3. Fabricate the Eg-GFET

The procedures for fabricating the Eg-GFET were similar to our previous work [23], with the only difference in the layout of the gate electrode, which is the coplanar Ag/AgCl electrode in Figure 1A. The main steps are listed below: (1) G-ink was printed by microelectronic printer on the clean PI substrate, and then dried at 70 °C and annealed at 250 °C for 4 h. (2) Silver ink was printed on the two sides of the channel as drain and source electrodes. (3) Ag/AgCl ink was printed near the source electrode. (4) The electrodes were dried at 70 °C, and the devices were encapsulated by silica gel except for the regions of the channel and the gate electrode.

### 2.4. Ions Detection

(1) A total of 50 μL EDC/NHS solution was added to the prepared Eg-GFET channel to activate the carboxyl group of the G-ink at room temperature for 30 min. (2) Then, 50 μL 1 μM calcium aptamer was incubated on Eg-GFET at 4 °C overnight. (3) The standard Ca^2+^ solution (1000 mg/L) was diluted to obtain solutions in different concentrations of 100, 10, 1, 10^−1^, 10^−2^, 10^−3^, 10^−4^ and 10^−5^ mg/L, respectively. These solutions from low to high concentrations were added to the surface of the as-prepared Eg-GFET Ca^2+^ sensor and incubated for 5 min at room temperature. After being rinsed, the sensors waited for measurement. (4) The Na^+^, Mg^2+^, Ba^2+^ and K^+^ ions solutions with a concentration of 10 mg/L were obtained by dissolving NaCl, MgCl_2_, BaCl_2_ and KI in deionized water, respectively. Similar to the detection of Ca^2+^, they were measured by Eg-GFET Ca^2+^ sensors.

### 2.5. Regression Modeling

The processes of studying the proposed regression model are listed as follows:

(1) Data pretreatment and data set establishment: the bias voltage and Δ*I_ds_/I*_(*ds*,0)_ were used as input, and logarithm of Ca^2+^ concentration was used as the label to compose the data set.

(2) Data set division: the data set was divided into training sets and testing sets, accounting for 80% and 20%, respectively. The Scikit-learn library in Python was used to divide the data into boxes to ensure that the data under all bias voltages could be evenly distributed between the training sets and the testing sets. Finally, the training sets contained 14,400 data. The testing sets have 3600 data.

(3) Model training: LR, SVR, DTR and RFR algorithms in the Scikit-learn library were, respectively, used to train regression models by using data in the training sets, during which fivefold cross validation was used in SVR, DTR and RFR algorithms.

(4) Model evaluation: the bias voltage and Δ*I_ds_/I*_(*ds*,0)_ in the testing sets were used as the input of the regression model, and the predicted value of Ca^2+^ concentration logarithm was obtained and compared with the true value to calculate the error distribution.

## 3. Results

### 3.1. X-ray Photoelectron Spectroscopy (XPS) Characteristic

**Figure 2:**
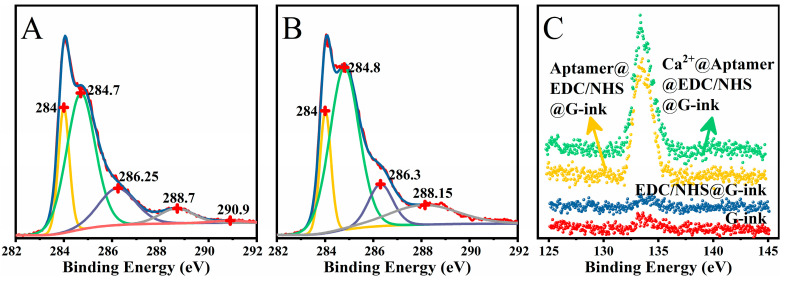
XPS Characterization: (**A**) the deconvoluted C1s spectra of G-ink film; (**B**) the deconvoluted C1s spectra of EDC/NHS treated G-ink (EDC/NHS@G-ink); (**C**) the P2p spectra of G-ink, EDC/NHS@G-ink, after aptamer immobilized (Aptamer@EDC/NHS@G-ink) and after dropped Ca^2+^ (Ca^2+^@Aptamer@EDC/NHS@G-ink).

We verified the functionalization strategy by using XPS, and the results are shown in Figure 2. The C1s spectra of the G-ink sample are shown in Figure 2A, where the peak at 288.7 eV belongs to the carboxyl group [24]. After EDC/NHS treatment, the peak at 288.7 eV disappears, and a new peak appears at 288.15 eV (in Figure 2B), which corresponds to the C-N-C [25]. This indicates that the carboxyl groups in G-ink samples are activated by EDC/NHS to form succinimide groups [26], which can be used to covalently connect to the amino-modified aptamers. In Figure 2C, the peaks of P element in the samples before the aptamer being modified are almost negligible, as shown by the blue and red data points. Conversely, there are obvious peaks at 133.6 eV in the aptamer-modified samples, as shown by the green and yellow data points. This suggests that the aptamers were successfully fixed on the surface of Eg-GFET, and the aptamers were still retained after being incubated with Ca^2+^ solution.

### 3.2. Electrical Properties of Eg-GFETs

**Figure 3:**
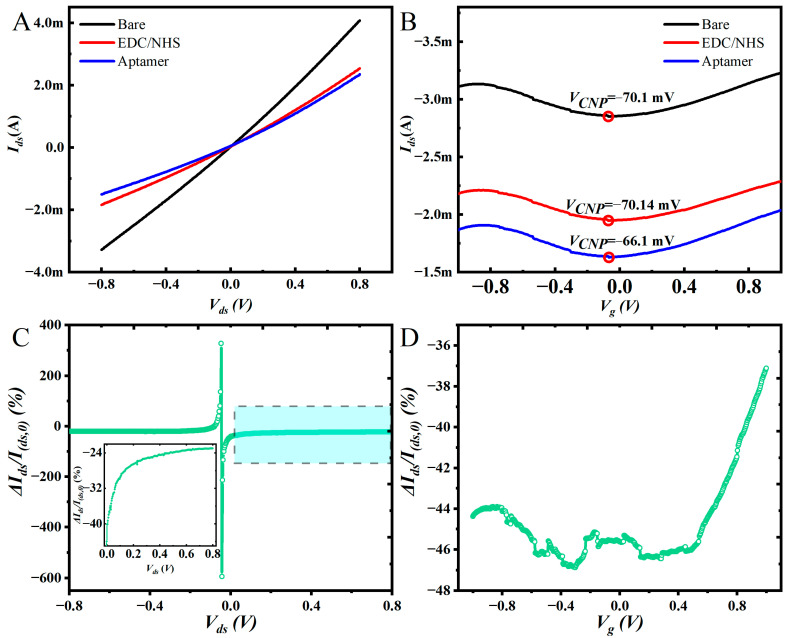
Output (**A**) and transfer (**B**) characteristics of bare Eg-GFET after being treated by the EDC/NHS treated and modified by aptamer. Δ*I_ds_/I*_(*ds*,0)_ changing along with *V_ds_* (**C**) and *V_g_* (**D**).

We measured the output and transfer curves of Eg-GFETs before and after being functionalized by the procedures depicted in Figure 1A. The output curves were measured when the voltage of *V_ds_* was scanned from −0.8 V to 0.8 V with the step of 3.2 mV and the value of *V_g_* was controlled at −0.5 V. The results are shown in Figure 3A, *I_ds_* of the Eg-GFETs decreased after each step of functionalization. Similarly, the transfer curves were measured when the voltage of *V_g_* was scanned from −1 V to 1 V with the step of 4 mV and the value of *V_ds_* was controlled at −0.8 V. The results are shown in Figure 3B, which indicates that *V_CNP_* of bare Eg-GFET is −70.1 mV. After EDC/NHS treatment, *V_CNP_* is shifted in the left direction to −70.14 mV, and the aptamer immobilization makes *V_CNP_* move right slightly to −66.1 mV due to the negative charges on the phosphate backbone of aptamer molecules [27]. Compared to the bare Eg-GFET, the transfer curves after being treated by EDC/NHS and modified by the aptamer are lowered. This may be caused by the reduced carriers’ concentration, which is consistent with the results of the output curve.

Normally, a constant working condition, i.e., the fixed *V_ds_* and *V_g_*, may be used in the following Ca^2+^ detection, but the measured variations of the transfer curves and the output curves indicate that the values of *I_ds_* are greatly dependent on the chosen *V_g_* or *V_ds_*. So, we measured the responses to Ca^2+^(10 mg/L) at different working conditions which is shown in Figure 3C,D. In Figure 3C,D, *I*_(*ds*,0)_ is the current value of the Eg-GFET modified aptamer, and *I_ds_* is the current value of the device incubated with Ca^2+^(10 mg/L); Δ*I_ds_* is *I_ds_* minus *I*_(*ds*,0)_. Figure 3C is obtained from output curves at each of *V_ds_*. It can be seen that the Δ*I_ds_/I_(ds_*_,0*)*_ changes sharply when *V_ds_* is close to 0 V and changes slightly when *V_ds_* is far from 0 V (Figure 3C). For example, the Δ*I_ds_/I_(ds,_*_0*)*_ is −23.02% when *V_ds_* is 0.8 V and −40.1% when *V_ds_* is 4.87 mV (inset of Figure 3C). There are sharp peaks in Figure 3C at −46 mV and −43 mV. The reason may be that *V_ds_* is close to zero which makes the potential along the channel almost zero and cannot drive carriers flow. *I_ds_* in this state is tiny; the weak changes of interfacial impendence caused by Ca^2+^ binding on aptamer can produce an acute current change rate. Figure 3D is obtained from the transfer curve at each of *V_g_*; it indicates that Δ*I_ds_/I_(ds,_*_0*)*_ decreases slightly from −44.37% to −46.36% when *V_g_* = −1–−0.4 V. It fluctuates around −46% in the range of *V_g_* = −0.4–0.4 V and increases almost linearly from −46% to −37.1% when *V_g_* = 0.4–1 V. Deductions about the curve in Figure 3D are proposed according to the mechanisms of Eg-GFET at each side of the Dirac point. At the left of the Dirac point, the hole is majority carriers, and the conformational changes of aptamer after reaction with Ca^2+^ cannot dramatically alter the carriers’ concentration. So, the left part in the curve of Figure 3D only has small variation. Conversely, the right part grows up gradually with the increasing of *V_g_*; it is coincident with the electron dominated working mechanism in the right side of the Dirac point because the negative charges on the aptamers’ phosphate backbone can induce positive holes in the channel which can have notable impact on the carrier’s concentration. Therefore, at the fixed *V_ds_*, because the reaction between aptamer and Ca^2+^ mainly influence the holes’ concentration, the relation of Δ*I_ds_/I*_(*ds*,0)_ with *V_g_* is placid in the left side and gradually increased in the right side.

Figure 3C,D show the bias voltages (*V_ds_, V_g_*) are the important factors influencing the response of Eg-GFET sensors. They also indicate that the selected working conditions have significant impact on the sensitivity of the sensors. So, single work conditions cannot fully reflect the performance of the sensor, but it is cumbersome to make calibration curves for all possible working conditions. We think machine learning can provide a solution to solve this problem by modeling the output curves or transfer curves to directly determine the ions’ concentration free from calibration curve.

### 3.3. Ca^2+^ Ion Test

**Figure 4:**
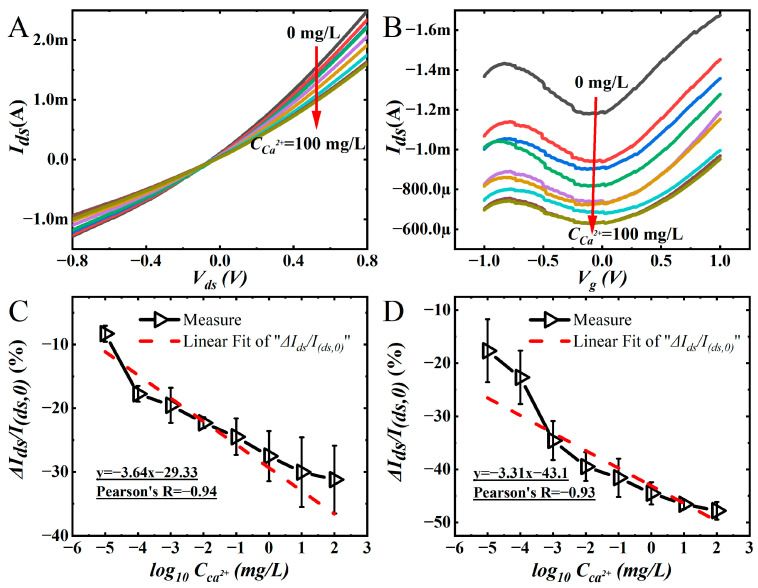
Sensor performance test: (**A**)the output curve of Ca^2+^ incubated on the surface of the sensor at different concentrations; (**B**) the transfer curve of Ca^2+^ incubated on the surface of the sensor at different concentrations; (**C**) the Δ*I_ds_/I*_(*ds*,0)_ at different $C_{Ca^{2+}}$ when *V_ds_* = 0.8 V on the output curve; (**D**) the *ΔI_ds_/I*_(*ds*,0)_ at Dirac point after incubation of Ca^2+^ solutions ($C_{Ca^{2+}}$ = 10^−5^–10^2^ mg/L).

The responses of the Eg-GFET Ca^2+^ sensors were measured with a gradually increasing concentration of Ca^2+^ ($C_{Ca^{2+}}$), as shown in Figure 4A,B. The slopes of the output curves decreased, and the transfer curves shifted down with the increase of $C_{Ca^{2+}}$. The descending output curves are caused by the decreased carriers due to the increased impendence at the interface of Ca^2+^ aptamer functionalized graphene and electrolyte solution. The transfer curves moved downward with a slightly left shift; it indicates the reaction between Ca^2+^ and aptamer mainly influence the interfacial impendence. It has almost no impact on the doping state in the graphene channel because of nearly unchanged *V_CNP_*. There is no doubt that the sensor is sensitive to Ca^2+^, but to further examine its sensitivity, the current change rate (Δ*I_ds_/I*_(*ds*,0)_) is calculated by using the data in Figure 4A,B.

The results of Δ*I_ds_/I*_(*ds*,0)_ vs. $C_{Ca^{2+}}$ are presented in Figure 4C,D. These are coincident with the traditional method, i.e., the voltage of *V_ds_* and *V_g_* are constant; the value of Δ*I_ds_/I*_(*ds*,0)_ could be used to determine $C_{Ca^{2+}}$. In Figure 4C, the values of Δ*I_ds_/I*_(*ds*,0)_ were obtained at fixed *V_ds_* (0.8 V) and *V_g_* (−0.5 V), *I_(ds,_*_0)_ is the current value of the Eg-GFET modified aptamer, and *I_ds_* is the current value of the device incubated at different $C_{Ca^{2+}}$, Δ*I_ds_* is *I_ds_* minus *I*_(*ds*,0)_. The linear fit line (the red dash line in Figure 4C) shows the sensitivity is 3.64% per Log_10_$C_{Ca^{2+}}$, and Pearson’s R is −0.94. In Figure 4D, *I_(ds,_*_0)_ is the current value at *V_CNP_* after aptamer modification, *I_ds_* is the current value at *V_CNP_* after incubation with different $C_{Ca^{2+}}$, the linear fit line (the red dash line in Figure 4D) shows the sensitivity is 3.31% per Log_10_$C_{Ca^{2+}}$, and Pearson’s R is −0.93. Accordingly, the different work conditions have an apparent effect on the sensitivity which agrees with the discussion about Figure 3C,D, so the traditional calibration method at fixed working condition is not enough to evaluate the sensors performance and determine the analyte concentration. It is therefore necessary to find some new method to solve this problem. More information about sensors’ selectivity is provided in the Supplementary Material.

### 3.4. Regression Algorithm Analyzes Output Curves and Transfer Curves

**Figure 5:**
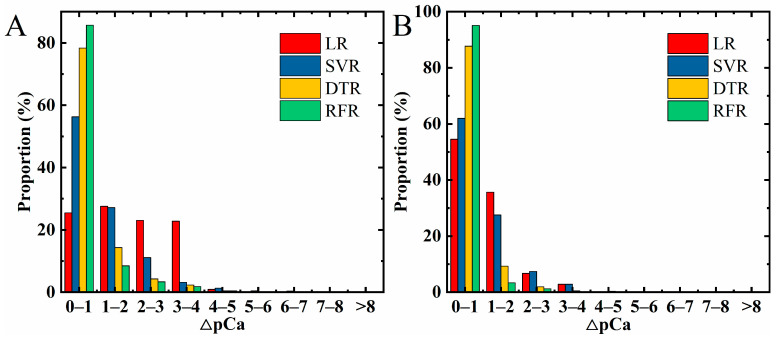
Evaluation results of regression models by using algorithms of LR, SVR, DTR and RFR: (**A**) regression models of output features; (**B**) regression models of transfer features.

To overcome the limitations of conventional data analyzation due to the fixed working condition, we attempted to establish regression models of output and transfer features by using machine learning algorithms, including LR, SVR, DTR and RFR. Then, the trained regression models can be applied to directly determine the analyte’s $C_{Ca^{2+}}$. Firstly, the models were obtained from 14,400 data in the training sets (as mentioned in Section 2.4), in which the bias voltages (*V_ds_*, *V_g_*) and *ΔI_ds_/I*_(*ds*,0)_ were used as input, and the logarithms of $C_{Ca^{2+}}$ were used as output. Then, the regression models were evaluated by using 3600 data in the testing sets. The proportions of absolute difference between predicted and real values are shown in Figure 5, in which ΔpCa is:

$$△\mathrm{pCa}=\left|{\mathrm{Log}}_{10}C_{Ca^{2+}}{}_{predicted}-{\mathrm{Log}}_{10}C_{Ca^{2+}}{}_{real}\right|$$

The figures indicate the degrees of preciseness which were produced by four different regression algorithms, i.e., LR, SVR, DTR and RFR. For the DTR and RFR, the highest columns are focused in the error range of 0–1, but for the LR and SVR, the columns are scattered in the ranges of 0–1, 1–2, 2–3 and 3–4, which means more data could be predicted correctly by using DTR and RFR than the other two.

Furthermore, for the output features, their errors in the range of 0–1 account for 25.47% (LR), 56.33% (SVR), 78.36% (DTR) and 85.72% (RFR) of 3600 testing data, as shown in Figure 5A, which indicate the RFR model has the highest accuracy. For transfer features (in Figure 5B), the errors in the range of 0–1 account for 54.58% (LR), 62% (SVR), 87.72% (DTR) and 95.11% (RFR) of 3600 testing data, which means the RFR model is also the best one. Moreover, the comparison of Figure 5A,B shows the transfer features may be more suitable for training the regression model of Eg-GFET sensors because of higher columns in the low error range. This result is consistent with other methods which fit the relation *V_CNP_* offset to the analyte’s concentration [28]. For the internal mechanism, we think it may be that the combination of Ca^2+^ and aptamer on the device surface causes not only the current decrease but also the Dirac point shift, which both lead to the transfer curves being more easily distinguished than output curves as evidenced by Figure 4A,B.

In addition, more evaluation indicators are presented in Table 1. Among the four algorithms, the best regression algorithm is RFR obtained from transfer curves, which has an MSE of 0.24, an RMSE of 0.49, an MAE of 0.21 and an R^2^ of 0.96. To sum up, the performance of the four algorithms can be ranked from best to worst as: RFR > DTR > SVR > LR. Compared to the regression models obtained by analyzing the output curves, the regression model obtained by analyzing the transfer curves is better.

### 3.5. Prediction of $C_{Ca^{2+}}$

**Figure 6:**
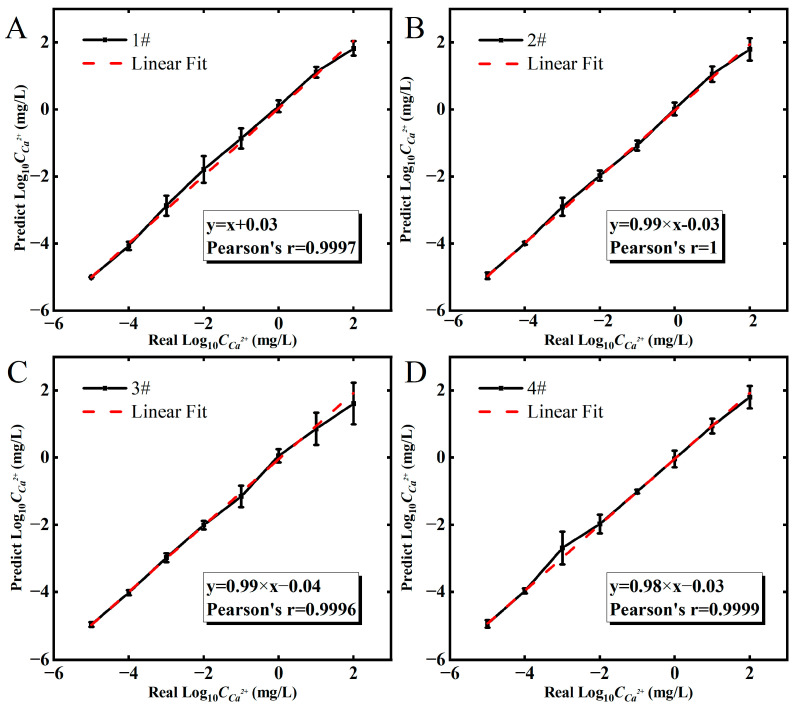
The results of $C_{Ca^{2+}}$ calibration-free determination for four individual Eg-GFET sensors named as 1# (**A**); 2# (**B**); 3# (**C**); 4# (**D**), respectively. The error bar represents the standard deviations for n measurements (n = 500).

By far, the RFR model from transfer features is confirmed to be better for determination of $C_{Ca^{2+}}$ in the platform of Eg-GFET sensors than the LR, SVR and DTR algorithms. As a result, the RFR model is applied to predict the tested $C_{Ca^{2+}}$, the relations of predicted $C_{Ca^{2+,}}$.Its real values are shown in Figure 6. For the four individual sensors, their predicted $C_{Ca^{2+}}$ are all in good linear relations (close to y = x) with real concentrations in the range of 10^−5^ to 10^2^ mg/L, as shown by the red dashed lines in Figure 6A–D. Pearson’s correlation coefficient is greater than 0.999, which means the RFR model possesses higher accuracy than the traditional method based on fixed working conditions. Pearson’s correlation coefficients are about −0.94 and −0.93 (in Figure 4C,D).

## 4. Discussion

In this paper, we described a new method to determine the analyte’s concentrations directly from the responding signal of an Eg-GFET Ca^2+^ sensor by using machine learning. It is found that the traditional calibration method at the fixed working condition for ascertaining $C_{Ca^{2+}}$ is not suitable because it cannot fully reveal the performance of the sensor. To avoid the trouble of multiple calibrations, four regression algorithms (LR, SVR, DTR and RFR) were studied for constructing models of Eg-GFETs’ output and transfer features. The regression models between bias voltage (*V_ds_*, *V_g_*), Δ*I_ds_/I*_(*ds*,0)_ and Ca^2+^ concentrations were trained and evaluated by using 14,400 and 3600 data, respectively. It was confirmed the transfer features can be fitted by the RFR algorithm best, according to the metrics of MSE, RMSE, MAE and R^2^. The determined $C_{Ca^{2+}}$ by using the RFR-based regression model of transfer features is in good linear relation with its real value. Generally, aiming at the inhomogeneity of Eg-GFET sensors at different working conditions, we put forward an approach for coupling machine learning with Eg-GFET sensors, which is beneficial for promoting the applications of the Eg-GFET sensor or something similar because it can not only provide an accurate result but also facilitate the use of the sensor due to independence from calibration.

## Figures and Tables

**Table 1:** Evaluation indicators of regression models obtained by LR, SVR, DTR and RFR algorithms.

	Algorithms	LR		SVR		DTR		RFR	
Metrics		Output	Transfer	Output	Transfer	Output	Transfer	Output	Transfer
MSE		6.62	1.68	2.41	1.53	1.06	0.54	0.84	0.24
RMSE		2.57	1.29	1.55	1.24	1.03	0.73	0.91	0.49
MAE		2.23	1.03	1.19	0.97	0.56	0.39	0.43	0.21
R2		0.03	0.75	0.65	0.77	0.84	0.92	0.88	0.96

## Data Availability

Data sharing not applicable.

## Supplementary Materials

The following supporting information can be downloaded at: https://www.mdpi.com/article/10.3390/s23010353/s1, Figure S1: The selectivity of the sensor.
