# Supplementary material for: Electrolyte-Gated Graphene Field Effect Transistor-Based Ca2+ Detection Aided by Machine Learning
Source: Sensors (Basel). 2022 Dec 29;23(1):353. doi: 10.3390/s23010353 (PMC9824237; doi:10.3390/s23010353)
Supplement: Supplementary file 1 [file sensors-23-00353-s001.zip › sensors-2100631-supplementary.pdf]

Supplemental material

Calibration-Free Ca^{2+} Detection by Lg-GFET Sensor and Machine Learning

In order to evaluate the selectivity of the sensors, we also tested the output and transfer curves after incubation with Ba^{2+}, K^{+}, Mg^{2+} and Na^{+} aqueous solutions (10 mg/L). The responding results are shown in Figure S1, in which $\Delta I_{ds}/I_{(ds,0)}$ was calculated by using the same method give in the manuscript (section 3.2). It can be seen that there are also responses to Ba^{2+}, K^{+}, Mg^{2+} and Na^{+}, but their $\Delta I_{ds}/I_{(ds,0)}$ valus are relatively lower than Ca^{2+}, indicating that the devices have a good selectivity for Ca^{2+}.

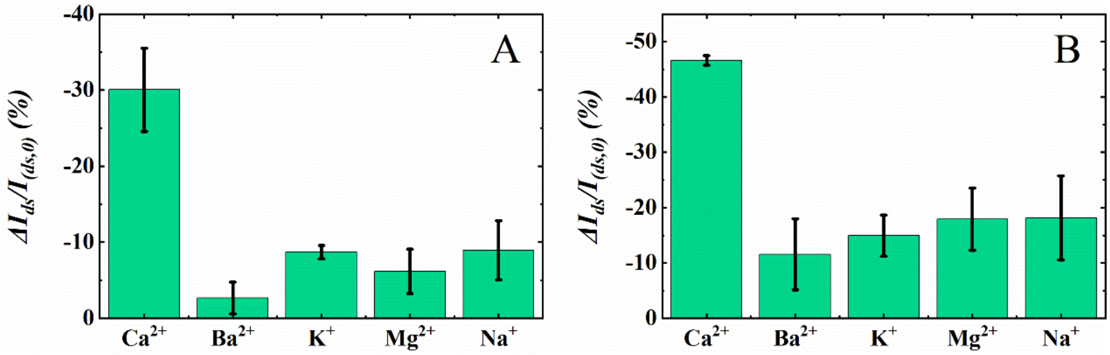

Figure S1. The selectivity of the sensor. (A) The calculated $\Delta I_{ds}/I_{(ds,0)}$ at V_{ds}=0.8V on the output curve which measured after Ba^{2+}, K^{+}, Mg^{2+} and Na^{+} solutions dropped. (B) The calculated $\Delta I_{ds}/I_{(ds,0)}$ at V_{CNP} on the transfer curve which measured after Ba^{2+}, K^{+}, Mg^{2+} and Na^{+} solutions dropped. The error bar represents the standard deviations (n=4).

The main code of training transfer curve to get LR, SVR, DTR and RFR models was shown in below, the code for training the output curve is similar.

1. Divide data set

```
#希望测试集能够代表整个数据集中不同电压时的输入
data["divide"]=pd.cut(data["Vg"],bins=10)  #bins取整数，即指定分箱的箱子个数
split=StratifiedShuffleSplit(n_splits=1,test_size=0.2,random_state=42)
for train_index,test_index in split.split(data,data["divide"]):
    strat_train_set=data.loc[train_index]
    strat_test_set=data.loc[test_index]
strat_train_set.to_csv(ml_path+"训练集.csv")
strat_test_set.to_csv(ml_path+"测试集.csv")
print("训练集个数",len(strat_train_set))
print("测试集个数",len(strat_test_set))
#数据集划分完成之后，我们将divide列删除
for set_ in (strat_train_set,strat_test_set):
    set_.drop("divide",axis=1,inplace=True)
# 复制数据集
data_process=strat_train_set.copy()
corr_matrix=data_process.corr()
print("各属性之间的相关系数",corr_matrix)
attributes = ["Vg", "IdsChg", "Con"]
# 训练集
data_ml_train_sample=data_process.drop("Con",axis=1)
data_ml_train_target=data_process["Con"].copy()
# 测试集
data_ml_test_sample=strat_test_set.drop("Con",axis=1)
data_ml_test_target=strat_test_set["Con"].copy()
# 打印测试集格式
print("测试集数量：",len(data_ml_test_sample))
```

2. LinearRegression

```
lin_reg=LinearRegression()
lin_reg.fit(data_ml_train_sample,data_ml_train_target)
joblib.dump(lin_reg,ml_path+"transfer_lin_reg.pk1")  #保存模型
transfer_lin_reg=joblib.load(ml_path+"transfer_lin_reg.pk1")#加载模型
Lin_Con_Predictions=transfer_lin_reg.predict(data_ml_test_sample)
lin_mse=mean_squared_error(data_ml_test_target,Lin_Con_Predictions)
lin_mae=mean_absolute_error(data_ml_test_target,Lin_Con_Predictions)
lin_r2=r2_score(data_ml_test_target,Lin_Con_Predictions)
MSE["线性mse"]=lin_mse
MAE["线性MAE"]=lin_mae
R2["线性R2"]=lin_r2
print("线性模型",lin_mse)
err=pd.DataFrame()
err[0]=abs(Lin_Con_Predictions-data_ml_test_target)
# 个数统计
x=["0","1","2","3","4","5","6","7",">8"]
num=[]
for i in range (0,9,1):
    num.append(((err[0]>=i)&(err[0]<i+1)).values.sum())
print("num的个数",len(num))
lin_y=[]
for i in num:
    lin_y.append(100*i/len(data_ml_test_sample))
print("lin_y",lin_y)
# color="red" 设置柱子颜色
plt.bar(x,lin_y,align="edge",color="red",alpha=0.5)
plt.xlabel("Abs(error)")
plt.ylabel('Proportion(%)')
plt.grid(True)
save_fig(sensor+"转移线性模型bar图") #先保存再显示
plt.show()
```

3. Support Vector Regression

```
# 支持向量回归
svr_param_grid=[
    {"kernel":['linear','poly','rbf']},
    {'degree':[1,3,5],'C':[0.1,1,5],'epsilon':[0.1,0.7,1]}
]
svm_reg=SVR()
svr_grid_search=GridSearchCV(svm_reg,svr_param_grid,cv=5,scoring='neg_mean_squared_error',
                             return_train_score=True)
svr_grid_search.fit(data_ml_train_sample,data_ml_train_target)
print("支持向量回归最合适的参数：",svr_grid_search.best_params_)
joblib.dump(svr_grid_search.best_estimator_,ml_path+"transfer_svr_reg.pk1")  #保存模型
transfer_svr_reg=joblib.load(ml_path+"transfer_svr_reg.pk1")#加载模型
svm_reg_Predictions=transfer_svr_reg.predict(data_ml_test_sample)
svm_reg_mse=mean_squared_error(data_ml_test_target,svm_reg_Predictions)
svm_reg_mae=mean_absolute_error(data_ml_test_target,svm_reg_Predictions)
svm_reg_r2=r2_score(data_ml_test_target,svm_reg_Predictions)
MSE["支持向量回归MSE"]=svm_reg_mse
MAE["支持向量回归MAE"]=svm_reg_mae
R2["支持向量回归R2"]=svm_reg_r2
parameter["支持向量回归参数"]=svr_grid_search.best_params_
print("支持向量模型mse",svm_reg_mse)
svm_reg_err=pd.DataFrame()
svm_reg_err[0]=abs(svm_reg_Predictions-data_ml_test_target)
num=[]
count_all=pd.DataFrame()
for i in range (0,9,1):
    element=(((svm_reg_err[0]>=i)&(svm_reg_err[0]<i+1))).values.sum()
    num.append(element)
print("num",num)
svr_y=[]
for i in num:
    svr_y.append(100*i/len(data_ml_test_sample))
print("svr_y",svr_y)
plt.bar(x,svr_y,align="edge",color="orange",alpha=0.5)
plt.xlabel("Abs(error)")
plt.ylabel('Proportion(%)')
plt.grid(True)
save_fig(sensor+"svr模型bar图") #先保存再显示
plt.show()
print("MSE",MSE)
```

4. Decision Tree Regression

```
df=pd.DataFrame()
dtr_param_grid=[
    {'max_depth':[1,5,10],'min_samples_split': [1,5,10]}]
decision_tree_reg=DecisionTreeRegressor()
dtr_grid_search=GridSearchCV(decision_tree_reg,dtr_param_grid,cv=5,scoring='neg_mean_squared_error',
                             return_train_score=True)
dtr_grid_search.fit(data_ml_train_sample,data_ml_train_target)
print("DTR最合适的参数：",dtr_grid_search.best_params_)
joblib.dump(dtr_grid_search.best_estimator_,ml_path+"transfer_dtr_reg.pkl")  #保存模型
transfer_dtr_reg=joblib.load(ml_path+"transfer_dtr_reg.pkl")#加载模型
decision_tree_Predictions=transfer_dtr_reg.predict(data_ml_test_sample)
decision_tree_mse=mean_squared_error(data_ml_test_target,decision_tree_Predictions)
decision_tree_mae=mean_absolute_error(data_ml_test_target,decision_tree_Predictions)
decision_tree_r2=r2_score(data_ml_test_target,decision_tree_Predictions)
MSE["decision_treemse"]=decision_tree_mse
MAE["决策树MAE"]=decision_tree_mae
R2["决策树R2"]=decision_tree_r2
parameter["决策树回归参数"]=dtr_grid_search.best_params_
df=pd.DataFrame()
df["test_target"]=data_ml_test_target
df["test_predictions"]=decision_tree_Predictions
df.to_csv(ml_path+sensor+"转移曲线决策树回归模型预测结果.csv")
print("决策树回归模型mse:",decision_tree_mse)
err1=pd.DataFrame()
err1[0]=abs(decision_tree_Predictions-data_ml_test_target)
#绘制bar图
num=[]
count_all=pd.DataFrame()
for i in range (0,9,1):
    element=(((err1[0]>=i)&(err1[0]<i+1))).values.sum()
    num.append(element)
print("num",num)
dt_y=[]
for i in num:
    dt_y.append(100*i/len(data_ml_test_sample))
print("dt_y",dt_y)
# edgecolor="blue"  设置柱子颜色
plt.bar(x,dt_y,align="edge",color="blue",alpha=0.5)
plt.xlabel("Abs(error)")
plt.ylabel('Proportion(%)')
plt.grid(True)
save_fig(sensor+"决策树模型bar图") #先保存再显示
plt.show()
```

5. Random forest regression

```
# 随机森林模型
rf_param_grid=[
    {'min_samples_split': [2,5,10]}
]
random_forest_reg=RandomForestRegressor()
rfr_grid_search=GridSearchCV(random_forest_reg,rf_param_grid,cv=5,scoring='neg_mean_squared_error',return_train_score=True)
rfr_grid_search.fit(data_ml_train_sample,data_ml_train_target)
print("RFR最合适的参数：",rfr_grid_search.best_params_)
joblib.dump(rfr_grid_search.best_estimator_,ml_path+"transfer_rfr_reg.pkl")  #保存模型
transfer_rfr_reg=joblib.load(ml_path+"transfer_rfr_reg.pkl")#加载模型
forest_reg_Predictions=transfer_rfr_reg.predict(data_ml_test_sample)
forest_reg_mse=mean_squared_error(forest_reg_Predictions,data_ml_test_target)
forest_reg_mae=mean_absolute_error(data_ml_test_target,forest_reg_Predictions)
forest_reg_r2=r2_score(data_ml_test_target,forest_reg_Predictions)
MSE["forest_reg_mse"]=forest_reg_mse
MAE["随机森林MAE"]=forest_reg_mae
R2["随机森林R2"]=forest_reg_r2
parameter["随机森林回归参数"]=rfr_grid_search.best_params_
print("随机森林模型mse：",forest_reg_mse)
err2=pd.DataFrame()
err2[0]=abs(forest_reg_Predictions-data_ml_test_target)
num=[]
count_all=pd.DataFrame()
for i in range (0,9,1):
    element=(((err2[0]>=i)&(err2[0]<i+1))).values.sum()
    num.append(element)
print("num",num)
rf_y=[]
for i in num:
    rf_y.append(100*i/len(data_ml_test_sample)) #
print("rf_y",rf_y)
plt.bar(x,rf_y,align="edge",color="green",alpha=0.5)
plt.xlabel("Abs(error)")
plt.ylabel('Proportion(%)')
plt.grid(True)
save_fig(sensor+"随机森林模型bar图") #先保存再显示
plt.show()
#总结
pd.concat((pd.DataFrame([MSE]),pd.DataFrame([MAE]),pd.DataFrame([R2]),pd.DataFrame([parameter]))
,axis=0).to_csv(ml_path+sensor+"转移曲线四种算法性能总结.csv")
output_result=pd.DataFrame()
output_result=pd.concat((pd.DataFrame(x),pd.DataFrame(lin_y),
pd.DataFrame(svr_y),pd.DataFrame(dt_y),pd.DataFrame(rf_y)),axis=1)
output_result.to_csv(ml_path+"转移曲线误差百分比总结.csv")
```
